# 200 years of KIT/TH Karlsruhe, 125 Years of Polymer Science in Karlsruhe: The Place Where Staudinger's Polymer Research Started

**DOI:** 10.1002/marc.202500331

**Published:** 2025-09-08

**Authors:** Manfred Wilhelm

**Affiliations:** ^1^ Karlsruhe Institute of Technology Karlsruhe Germany

**Keywords:** Karlsruhe, Karlsruhe Institute of Technology (KIT), Polymer Science, Staudinger

## Abstract

Within this special issue we would like to celebrate 200 years of the Karlsruhe Institute of Technology (KIT) and the former Technical University Karlsruhe/Germany. The Technical University Karlsruhe served, according to the first president of MIT, William Barton Rogers, as the role model for the planned MIT in Boston/USA after he visited Karlsruhe. All authors of this special issue of *Macromolecular Rapid Communications* have been or are still active in Karlsruhe. Prof. Michael Meier, Prof. Patrick Théato and I are very thankful for these state‐of‐the‐art contributions in polymer science. It might not be well known that the foundation of polymer science has very strong roots in Karlsruhe. Therefore, the intention of this short historical article is to display this rich history and especially mention people and research groups who worked in Karlsruhe in the area of polymer science over the last approximately 125 years. The history of polymer science can be found in related textbooks.

## Abstract 2

Several aspects of the history of polymer science in Karlsruhe are presented. Over the last 125 years, very famous polymer scientists worked as professors in Karlsruhe, for example: Hermann Staudinger, Leo Ubbelohde, Hermann Franz Mark, and Werner Kuhn. These and other researchers are portrayed. Scientists working in Karlsruhe contributed substantially to the development of polymer science.

Within this special issue, we would like to celebrate 200 years of the Karlsruhe Institute of Technology (KIT) and the former Technical University Karlsruhe/Germany. The Technical University Karlsruhe served, according to the first president of MIT, William Barton Rogers, as the role model for the planned MIT in Boston, USA [1], after he visited Karlsruhe.

All authors of this special issue of *Macromolecular Rapid Communications* have been or are still active in Karlsruhe. Prof. Michael Meier, Prof. Patrick Théato, and I are very thankful for these state‐of‐the‐art contributions in polymer science. It might not be well known that the foundation of polymer science has very strong roots in Karlsruhe. Therefore, the intention of this short historical article is to display this rich history and especially mention people and research groups who worked in Karlsruhe in the area of polymer science over the last approximately 125 years [2]. The history of polymer science can be found in related textbooks [3, 4, 5].

The first polymerization of styrene might have taken place in Karlsruhe in or before 1900 in the group of Carl Engler and was published by himself and his assistant A. Kronstein between 1897 and 1902 [3, 4, 5, 6]. Later, the entire Ph.D. thesis of Mr. Lautenschläger (finished 1913) focused on polymerization. Very famous polymer scientists, specifically Hermann Staudinger, Leo Ubbelohde, Hermann Mark, and Werner Kuhn, all worked as professors in Karlsruhe, and contributed substantially to polymer science in these early days. Let us start from these early days and have a look at the individual scientists and research groups.

The first person in Karlsruhe who can be related to polymer science is Prof. Carl Engler (*1842–†1925), who held a chair of Chemical Technology. His research had a focus on mineral oils, and he developed a viscosimeter, the “Engler viscosimeter”, to characterize mineral oils [7]. These mineral oils often contained molecules with double bonds. He was further interested in the polymerization of styrene and, more generally, molecules containing C═C double bonds. Carl Engler was also a board member of BASF since 1903 and acted as a mentor for two young and very ambitious professors around 1910 at Karlsruhe: Fritz Haber, born 1868, and Hermann Staudinger, born 1881. Prof. C. Engler fostered Staudinger's relations to industry, specifically to BASF, and introduced him to polymerization. Hermann Staudinger, on the other side, helped Prof. C. Engler, already 71 years of age at the time, by supervising Ludwig (in short: Leo) Carl Lautenschläger during his Ph.D. to polymerize olefinic monomers. The polymerizations were performed both with and without peroxides as apparent initiators. Lautenschläger studied the kinetics of these polymerization reactions. According to the university academic calendar from 1910/11, Engler and Staudinger also jointly organized a weekly chemical colloquium for students [8].

In 1910, Leo Ubbelohde (*1876–†1964) conducted a habilitation in Chemical Technology in Karlsruhe [9] as Carl Engler had asked him to move from Berlin to Karlsruhe [10]. Habilitation is the German equivalent of an assistant professorship conducted under the supervision of a full professor, in his case Prof. Hans Bunte. Leo Ubbelohde was afterwards assistant and lecturer (German: *Privatdozent*) and received a professor's position in 1911 in Technical Chemistry in Karlsruhe, and took a position in 1933 at TU‐Berlin as a professor since others were forced out. His science was centered around petroleum. He is very well known for the Ubbelohde viscometer, which is still in use today in many laboratories around the world to study low viscosity solvents and dilute polymer solutions. At KIT, we still use it within our current polymer laboratory course. The experimental beauty is to dissolve a known mass of polymer and measure, with a simple stopwatch, the time needed for a specific volume of the solution to pass through a defined capillary, using a setup that compensates for pressure changes. The increase in the solution viscosity, as compared to the pure solvent, is then calculated from the elapsed time and allows the determination of the molecular weight of the dissolved polymer according to the Mark–Houwink equation. What is less known about Leo Ubbelohde is that in 1933, he signed up for the Nazi Party, officially the National Socialist German Workers' Party (German: Nationalsozialistische Deutsche Arbeiterpartei or NSDAP). He wanted to reshape the organization of chemists working in academia and industry within Germany toward the Nazi ideology [11]. He had discussions with Adolf Hitler about restructuring academia and specifically chemistry. In 1940 he had to retire early as a result of conflicts with the government [10]. Ubbelohde was earlier very active in the Red Cross at Karlsruhe, and took care of unemployed people after WW I. His students but also French and British colleagues praised him in a book from 1952 in the highest notation on the occasion of his 75th birthday [10]. He has received numerous international recognition of his work: Traveling the USA, president Theodor Roosevelt received him in 1908 at the White House; in 1932, he became honorary member of the “Institution of Petroleum Technologies” in London as the first German; in 1937 became honorary member of the Association Francaise des Techniciens du pétrole, Paris as the only German member; in 1938, he became member of the “*Ständiger Rat der Welt‐Erdöl Kongresse*”, London as the only German member. After WW II, already in 1950, he became an honorary fellow of the “Institution of Petroleum Technologies” in London as the first German [10].

The most well‐known polymer scientist from Karlsruhe is Hermann Staudinger (*1881–†1965) [6, 12, 13, 14, 15, 16]. He introduced the concept of macromolecules and received the Nobel Prize in chemistry in 1953 for his discoveries in the field of macromolecular chemistry. Staudinger was first trained as an organic chemist in Halle/Germany. In 1907, Staudinger finished his habilitation in Strasbourg, where he focused on organic chemistry, and his research included the study of ketenes, diazo compounds, and the use of oxaylchloride, just to give some examples. Directly in 1907, he received his first professorship in Karlsruhe. In 1912, he took a chaired professor position at ETH Zurich, Switzerland, and finally moved to Freiburg, Germany, in 1926. The influence of Carl Engler, from 1907 on, first led him to synthesize butadiene and isoprene, and thus led to the work on unsaturated monomers. The chemical reactivity of these two monomers, but also limonene, myrcene, and styrene, just to mention other investigated compounds, was very interesting to him. The Ph.D. thesis of Ludwig Carl Lautenschläger, finished 1913, was formally supervised by Prof. C. Engler [17]. Besides Engler, only H. Staudinger is acknowledged in the Ph.D. thesis of Lautenschläger [18]. Ludwig Carl Lautenschläger had a background as a pharmacist and worked, previously to his Ph.D. work, at the Lion pharmacy (*Löwenapotheke*), still a working pharmacy in 2025 in 76133 Karlsruhe, Kaiserstr. 72. This joint work between Staudinger and Engler was not published until 1931 [17]. The rather late contact between Lautenschläger and Staudinger became helpful for Staudinger in Freiburg. Lautenschläger was involved in the Nazi regime, running an important company for warfare (German: *Wehrwirtschaftsführer*) at IG Farben Hoechst [19]. In 1933, Lautenschläger wrote a letter of recommendation for Staudinger during his time in Freiburg, where Staudinger was almost pushed out of office due to a dispute with the new university president Martin Heidegger, a famous philosopher and strong Nazi supporter [19, 20]. Please note that at the time of Staudinger in Karlsruhe (1907–1912), Fritz Haber was an appointed professor, and Leopold Ruzicka was a Ph.D. student of Staudinger. All three scientists performed research in the same faculty at Karlsruhe, and all of them received, on independent topics, a Nobel prize. To conclude, during his time in Karlsruhe, Staudinger got familiar with three new aspects: polymerization, viscosity of polymer solutions, and contact with industry.

Prof. Egon Elöd (*1891–†1960) is less well known in the polymer community. He received his Ph.D. in 1914 with Engler and conducted his habilitation in 1924 in Karlsruhe. From 1925 on, he was a professor of textile chemistry and tannery chemistry. Egon Elöd was, from 1949 on, the first president of the German association of textile chemists and colorists, abbreviated in German as VTCC (German: *Verein der Textilchemiker und Coloristen e.V*.). Since 1977, this association awards the Egon Elöd prize to the best scientific publication in this field [21, 22]. One of his Ph.D. students in Karlsruhe was Helmut Zahn (*1916–†2004, Ph.D. 1940), who worked on textile chemistry and additionally became famous for the first synthesis of insulin in 1963. Helmut Zahn was from 1952 on the first director of the *Deutsches Wollforschungsinstitut*, DWI, in Aachen, which is still a very important institute in polymer science in Germany.

The name Hermann Franz Mark (*1895–†1992, engl.: Herman Francis Mark) is familiar to many polymer scientists [3, 4, 5]. Less known is his relation to Karlsruhe. Hermann Mark originated from Vienna, where he earned a Ph.D. in chemistry and taught Pauling about X‐rays and did experiments for Einstein. Fritz Haber invited him to work in Berlin at the Kaiser‐Wilhelm‐Institute, the predecessor of the Max‐Planck‐Institute. Due to an offer from Kurt Heinrich Meyer, he worked at the IG Farben in Ludwigshafen from 1927 to 1932, applying X‐ray diffraction to macromolecules and helped the company to understand and commercialize polystyrene, polyvinylchloride, and polyvinyl alcohol. To foster relations between BASF and a nearby university, Karlsruhe and Ludwigshafen are only 70 km apart, Hermann Mark finished his habilitation in Karlsruhe in 1927 and held an extraordinary professorship thereafter. The father of Hermann Mark was Jewish and had converted to Christianity upon marriage. Hermann Mark returned to Vienna at the start of the Nazi dictatorship in 1933. In 1938, he had to flee again and finally settled in New York in 1940, where he founded the Institute of Polymer Research at the Polytechnical Institute in Brooklyn, New York. Mark and especially Meyer are known for a heavy dispute with Staudinger related to the type of bonding, viscosity, and the size of the polymers within polymer segments: covalent in the view of Staudinger, Van der Waals in the view of Meyer and Mark. This dispute was finally settled externally around 1935, as the editor of a journal did not want to continue the public and very personal argumentation and dispute between Mark and Meyer on one side and Staudinger on the other side [23]. Scientifically, they were not far apart, it was also an argumentation about citations and priority, where Staudinger did not avoid an argument to be recognized as the person who owns priority in polymer science [6, 23]. Mark accepted Staudinger's argumentation and experimental proof that the binding had to be covalent and that molecules are larger than first thought. It might be worth mentioning that Prof. Helmut Ringsdorf, the last diploma student of Hermann Staudinger, did his postdoc in the group of Prof. Mark at Polytech. Ringsdorf and Staudinger knew each other very well, as Ringsdorf took the notes for his book “Arbeitserinnerungen” [6]. Ringsdorf assisted Staudinger during his visit to Brooklyn around 1961 and told me that Staudinger still showed at his lecture in Brooklyn, rigid wooden sticks as his models for polymer conformation. People were politely listening, but as Ringsdorf observed, time had moved on, the foundation was accepted, and new topics in polymer science had appeared [24].

Werner Kuhn (*1899–†1963) [25, 26, 27, 28] is well known in polymer science for the concept of the Kuhn length, a measure of persistence and a statistical quantity towards an ideal state. Werner Kuhn was Swiss by nationality and completed a Ph.D. at the ETH Zürich and a postdoc with Niels Bohr. Werner Kuhn was an associate professor in Karlsruhe from 1930 to 1936 and published in Karlsruhe his famous work: “*Über die Gestalt der fadenförmigen Moleküle in Lösungen*” [29], translated to “On the shape of strand‐like molecules in solution” in 1934. Kuhn had an extremely broad scientific scope and multiple interests. He was working on the statistics of polymer chains, with or without flow, but also on isotope enrichment. The Kuhn‐Martin process is still in use for ^235^U enrichment. Further scientific topics were dialysis, the structure of the Earth's crust, and optical activities of molecules. Like Hermann Mark, but less intense, Werner Kuhn was also involved in a dispute with Staudinger. This dispute was related to the random statistics of the polymer chains in solution, as foreseen by Werner Kuhn. The random statistics and conformational freedom of each covalent carbon–carbon bond along the polymer were not accepted by Staudinger at all at the time. Staudinger believed in rigid, covalent sticks to represent a polymer molecule. The dispute was obvious when Ph.D. students, often after a beer or two, were singing in Freiburg: *“Die Kuhnschen Knäuel sind uns hier ein Gräuel”*, which translates into “the beads (i.e., statistical segments) of Kuhn are a horror to us”. The two cities of Karlsruhe and Freiburg are about 100 km apart, and a direct train connection existed already at the time. Werner Kuhn left Karlsruhe for Kiel (1936) and later to Basel (1939), where he eventually became president of the university. In Basel, he first returned to polymer science and published, for example, work on poly(acrylic acid) as a model for muscles and worked on entropy‐based elasticity of entangled or cross‐linked polymers. In his later times, Staudinger agreed on the description Werner Kuhn had proposed: Polymer bonds are carbon–carbon covalent bonds that are rather freely rotating in solution at room temperature. This conformational freedom can be described by statistical means. Sometimes confusion arises between Werner Kuhn and Hans Kuhn (*1919–†2012, later director at the Max‐Planck Institute in Göttingen, Germany). Hans Kuhn was a former Ph.D. student of Werner Kuhn. Hans Kuhn wrote several articles related to his former supervisor, yet, these two persons are not related by family.

Friedrich August Henglein (*1893–†1968) studied chemistry in Karlsruhe, finished his Ph.D. 1919 in Heidelberg, conducted a habilitation in 1922 in Danzig, and held a professorship for Technical Chemistry in Karlsruhe from 1934 until 1961. He succeeded Prof. Paul Askenasy, who was forced to leave as he was Jewish. It is very interesting to read in the Karlsruhe University calendar [8] from 1910/11 that Askenasy held a lecture titled “*Bau und Betrieb der Accumulatoren*” that translates into “Building and modes of batteries,” a topic that KIT is worldwide recognized for 115 years later. Friedrich August Henglein joined the combat group of the German architects and engineers *(“Kampfbund Deutscher Architekten und Ingenieure”*) in 1933. From 1944 on, he was a member of the board of Staudinger's *Journal für Makromolekulare Chemie*, later renamed as *Die Macromolekulare Chemie*, and currently known as *Macromolecular Chemistry and Physics* (Wiley‐VCH). Henglein had close personal relations with Staudinger, they visited each other frequently. In 1950, Henglein wrote a letter to his faculty at Karlsruhe to suggest an honorary Ph.D. for Staudinger on the occasion of the 125th birthday of the Karlsruhe Technical University [30]. Staudinger kept close relations with Karlsruhe and presented his work nearly every year in a colloquium.

Bruno Vollmert (*1920–†2002) was a pilot in World War II and was shot down, resulting in large scars to his face [31]. After World War II, he conducted a Ph.D. in Karlsruhe in 1946 and a habilitation in 1951. From 1962 on, he was a professor in Karlsruhe with a focus on polymer science. Until 1986, he remained director of the Institute for Polymer Chemistry in Karlsruhe [2]. His research was focused on the synthesis, characterization, and reactions of macromolecules, including microgels and macrocycles. He had a scientific dispute with Prof. Manfred Eigen (Nobel prize 1967) on the kinetics and influence of polymer molecules towards the development of life. Vollmert wrote a book on this topic: “*Das Molekül und das Leben”* (The molecule and life) [32]. Within the German polymer community, Prof. Vollmert was very well known for his five‐volume textbook on polymer science, which was translated into several languages, including English and Chinese [31, 33]. A former technician of the author's group, Mr. W. Arbogast, who worked 52 years at KIT from 1968 till 2020, told the story that Paul Flory once honored Prof. Vollmert by saying that he, Flory, wrote with his textbook, “*Principles of Polymer Chemistry*” the bible of polymer science, but Bruno Vollmert wrote the five books of Moses, the Law [31].

In 2004, Prof. M. Ballauff and S. Höger arranged a fusion of the Institute of Technical Chemistry and the Institute of Polymer Chemistry, resulting in a single entitled Institute for Chemical Technology and Polymer Chemistry, ITCP, from then on.

The Professors who worked after Bruno Vollmert in Karlsruhe in the field of Polymer Science are: M. Ballauff (1990 ‐ 2003), synthesis and Physical Chemistry of colloids; H. Nimz (1968–1983), polymeric natural materials, lignin; D. Schlüter (1991–1992), synthesis, 2D materials; G. Wenz (1993–2002), synthesis, cyclodextrins, S. Höger (2002 ‐ 2006), shape persistent macromolecules; C. Barner‐Kowollik (2008–2016), controlled radical synthesis, molecular architectures, kinetics; M. Wilhelm (since 2006), anionic synthesis, method development, combined methods in rheology and SEC; M. Meier (since 2010), renewable polymer, sustainability, sequence‐definition; P. Théato (since 2017), post‐polymerization modification, polymer synthesis, sulfur based polymers.

For me personally, it is still hard to imagine the personal tension in a rather small faculty around the years 1933 where strong supporters of the new dictatorial regime, i.e. Henglein or Ubbelohde, were in close contact to current or former members and colleagues from the same chemistry faculty like Fritz Haber, Paul Askenasy and Hermann Mark, all of whom were considered Jewish and forced to leave.

I would like to close with a Figure taken from a special Simplicissimus brochure for Staudinger [33]. This brochure is dated July 12, 1912, and is dedicated to Staudinger on the occasion of his departure from Karlsruhe to ETH Zurich, and was drawn by his students. Shown is a humorous poem concerning the polymerization of butadiene to obtain synthetic rubber (in German: *Kautschuk*). This figure is a further, clear indication that Staudinger's research on polymers began in Karlsruhe. The motto stated in this figure is: To make rubber (*Kautschuk*) is not hard, but butadiene is hard to make (Figure 1).

**FIGURE 1:**
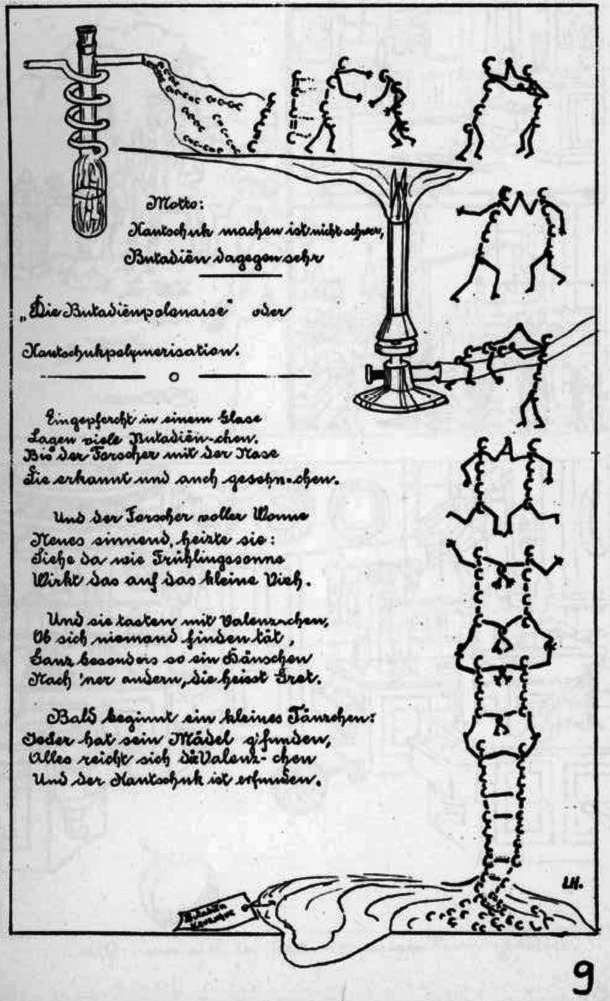
Butadiene polymerizes to rubber, as shown in the goodbye brochure from the students of Staudinger in the year 1912 on the occasion of his new position and departure for ETH Zurich [34].

## Conflicts of Interest

The authors declare no conflicts of interest.

## Data Availability

The author has nothing to report.

## References

[1] Information displayed in building 11.21, lecture hall room nr. 006 at KIT, campus south, Karlsruhe, also: Life and Letters of William Barton, Rogers, 2 Volumes, ed. E. Rogers u and W. T. Sedgwick 1 (2017): 216. 1896.

[2] “History of Polymer Chemistry in Karlsruhe,” Karlsruhe Institute of Technology, https://www.itcp.kit.edu/wilhelm/english/439.php.

[3] H. Morawetz , Polymers, The Origins and Growth of a Science, (John Wiley and Sons, 1995).

[4] Y. Furukawa , Inventing Polymer Science, Staudinger, Carothers, and the Emergence of Macromolecular Chemistry, (University of Pennsylvania Press, 1998).

[5] R. I. Tanner and K. Walters , Rheology: An Historical Perspective, (Elsevier, 1998).

[6] W. Jost , “H. Staudinger: Arbeitserinnerungen. Dr. Alfred Hüthig Verlag, Heidelberg 1961. 335 Seiten. Preis: DM 28,—,” Buchbesprechung 66 (1962): 77.

[7] C. Engler , Die Neueren Ansichten über die Entstehung des Erdöls, (Verlag für Fachliteratur, 1907): 25.

[8] Fridericiana , Grossherzoglich Badische Technische Hochschule zu Karlsruhe, Programm für das Studienjahr 1910 1911, Karlsruhe, Buchdruckerei von Malsch und Vogel 1910, KIT Archive Nr. 28014.

[9] R. E. Oesper , “Leo Ubbelohde,” Journal of Chemical Education 30 (1953): 414.

[10] L. Ubbelohde , S. Wirken , and K. G. Braun , KIT Library Signature: V A 1292, Written by His Students, Colleagues and Friends (KIT , 1952).

[11] H. Maier , Chemiker im “Dritten Reich”, (Wiley VCH, 2015).

[12] V. Percec , ed., Hierarchical Macromolecular Structures: 60 Years After the Staudinger Nobel Prize 1, Advances in Polymer Science, (Springer, 2013): 2013.

[13] R. Mühlhaupt , “Hermann Staudinger und der Ursprung der Makromolekularen Chemie,” Angewandte Chemie 116 (2004): 1072.

[14] H. Ringsdorf , “Hermann Staudinger und die Zukunft Der Polymerforschung Jubiläumsfeiern—Selbstbestimmter Anlass Kultureller Frömmigkeit,” Angewandte Chemie 116 (2004): 1082–1095.

[15] D. Braun , “Der Lange Weg Zum Makromolekül,” Chemie in Unserer Zeit 46 (2012): 310–320.

[16] A. Steinhofer , “Das Portrait: Hermann Staudinger,” Chemie in Unserer Zeit 4 (1967): 122–125.

[17] L. C. Lautenschläger , “Autooxydation und Polymerisation ungesättigter Kohlenwasserstoffe,” (Ph.D. Thesis, Großherzoglich Badischen Technischen Hochschule Fridericiana zu Karlsruhe 1913).

[18] L. Lautenschläger and H. Staudinger , “Über Hochpolymere Verbindungen, 51 Über Polymerisation und Auto‐Oxidation,” Liebigs Annalen Der Chemie 488 (1931): 1.

[19] E. Faupel , Chemie in Unserer Zeit 44 (2010): 396.

[20] C. C. Priesner , “Hermann Staudinger und die Makromolekulare Chemie in Freiburg. Dokumente Zur Hochschulpolitik 1925–1955,” Chemie in Unserer Zeit 21 (1987): 151–160.

[21] M. Rauch , Private Communication (Current President of VTCC, VDTF) (Applied University).

[22] Verband Deutscher Textilfachleute, https://www.vdtf.de/.

[23] C. Priesner , H. Staudinger , H. Mark , and K. H. Meyer , Thesen Zur Größe und Struktur der Makromoleküle, (Verlag Chemie Weinheim, 1980).

[24] H. Ringsdorf , Private Communication.

[25] H. Kuhn , “Das Portrait: Werner Kuhn (1899–1963),” Chemie in Unserer Zeit 19 (1985): 86–94.

[26] R. E. Oesper , “Werner Kuhn,” Journal of Chemical Education 27 (1950): 416.

[27] H. Kuhn , “Warner Kuhnd̊. (1899‐1963),” Helvetica Chimica Acta 47 (1964): 689–695.

[28] H. Kuhn , “Leben und Werk von Werner Kuhn 1899‐1963,” Chimia 38 (1984): 191.

[29] W. Kuhn , “Über die Gestalt Fadenförmiger Moleküle in Lösungen,” Kolloid‐Zeitschrift 68 (1934): 2.

[30] KIT archive Nr. 25003, letter dated 3.5.1950.

[31] W. Arbogast , Private Communication.

[32] B. Vollmert , Das Molekül und das Leben: Vom makromolekularen Ursprung des Lebens und der Arten: was Darwin nicht wissen konnte und Darwinisten nicht wissen wollen (German Edition), (Rowohlt, 1985).

[33] B. Vollmert , Grundriss Der Makromolekularen Chemie, Band 1–5, (E. Vollmert Verlag, 1988).

[34] KIT archives Nr. 28002/461, dated 12 July 1912. The scan of the whole brochure is available from the author.

